# Mechanical Birth Injuries: A Comprehensive Five-Year Analysis From a Tertiary Care Hospital

**DOI:** 10.7759/cureus.58997

**Published:** 2024-04-25

**Authors:** Duriya Rehmani, Anum Aziz, Ayesha Malik, Amir Raza, Alyna Khan

**Affiliations:** 1 Obstetrics and Gynaecology, Aga Khan University, Karachi, PAK; 2 Obstetrics, Aga Khan University, Karachi, PAK; 3 Obstetrics and Gynaecology, Aga Khan University Medical College, Karachi, PAK

**Keywords:** instrumental delivery, fracture at birth, emergency cesarean, shoulder dystocia, birth trauma, birth injury

## Abstract

Background: Birth injury or birth trauma refers to physical damage or trauma that occurs to a newborn during the birthing process. To ensure continuous care and improve neonatal outcomes, it is crucial to know the incidence, types, relation to the mode of delivery, and their management.

Methodology: This is a retrospective cohort study conducted at Aga Khan University Hospital, Pakistan from January 2018 to December 2022. Neonates aged from birth to 28 days of life identified to sustain any form of mechanical birth injuries were included. Data analysis was done using SPSS version 19 (IBM Corp., Armonk, NY).

Results: In the last five years, 51 mechanical birth injuries were found among 27,854 deliveries, which accounts for one in 546 births with an overall prevalence of 0.001%. Out of the total mechanical birth injuries, 12 (23.5%) were noticed in spontaneous vaginal delivery, six (11.8%) had instrumental delivery, and 33 (64.7%) patients had cesarean sections. More birth injuries were noticed in emergency cesarean section as compared to vaginal deliveries. There were 40 babies (78%) with soft tissue injuries, seven (14%) had musculoskeletal injuries/fractures, two (4%) babies had intracranial bleeding, and two (4%) had fractures along with intracranial bleeding. There was no mortality reported among these neonates.

Conclusion: The overall rate of birth injuries was significantly lower as compared to other low and middle-income countries. Most of the birth injuries were soft tissue injuries in patients with cesarean sections. The rate of birth injury did not show any association with the time of delivery. More frequent obstetric emergency drills would improve complications associated with shoulder dystocia.

## Introduction

Birth injury or birth trauma refers to physical damage that occurs to a newborn during the birthing process due to the natural forces of labor or because of obstetric interventions [1]. It can range from minor skin lacerations to major life-threatening events, which can cause severe morbidity or mortality and can have long-term effects on the lives of families and caretakers [2]. Mostly, these birth injuries do not require any follow-up and resolve spontaneously, but in some cases, immediate interventions are required [3]. However, the anticipation of birth trauma by the labor room attending team and early identification by the pediatric staff is crucial for improving perinatal outcomes [4].

There are various risk factors responsible for birth injuries that can occur due to a variety of reasons, including prolonged labor, abnormal fetal presentation, maternal health conditions, birth complications, and medical negligence. A study conducted in 2022 in Ethiopia concluded that primiparity, lack of education, and poor antenatal care are the important predisposing factors contributing to birth injuries [5]. Though literature is available on identified risk factors for birth injuries, the data regarding the prevalence of birth injuries and associated risk factors in resource-limiting settings in Asia are sparse. Graham et al. have identified the relationship between birth weight and birth trauma [6]. Instrumental delivery also stands out as one of the recognized risk factors for birth trauma [7].

Birth injuries are classified as minor or major, but data on the classification of birth injuries are scarce. Pressler in 2008 classified major injuries according to the type of tissue involved, how it has occurred, and the relationship of injury to long-term outcomes [8]. Major birth injuries include intracranial hemorrhage, peripheral nerve injury, and fractures, with clavicular and femur fractures and brachial plexus injury being the most common ones [7,8].

To ensure continuous care and improved neonatal outcomes, it is crucial to know the incidence, types, and relation to the mode of delivery, and early identification and their management are of immense importance. This can lead to enhanced patient care and improvement in the outcomes. There is a dearth of data on this subject as there is no proper portal of documentation and there is inadequate reporting of these injuries. Our study aims to assess the rate of birth injuries, types, and associated obstetric factors for newborns delivered at a tertiary care hospital in the last five years.

## Materials and methods

This five-year retrospective cohort study was conducted at the Department of Obstetrics and Gynecology, Aga Khan University Hospital, Pakistan. Neonates aged 0-28 days born, identified to sustain any form of mechanical birth injury ranging from minor lacerations to major trauma were included during the study period from January 2018 to December 2022. Neonates with a diagnosis of birth asphyxia, hypoxic ischemic encephalopathy, major congenital anomalies, or tumors were excluded. After receiving an exemption letter from the Ethical Review Committee of the Aga Khan Hospital, this study was initiated. Data were extracted from the Labor Room Management System, the Hospital's Adverse Event Management System, and the Neonatal Birth Registry System of the Aga Khan University Hospital using non-probability purposive sampling techniques. Maternal and neonatal demographics, type of birth injury, and associated factors of birth injury were recorded on a pre-structured proforma.

This is a retrospective analysis; therefore, informed consent is not required as there is no direct interaction with patients. The data were gathered by the team using a pre-designed study proforma and complete anonymity was maintained wherein the information was secured under a lock and key and password-protected files.

The data analysis was performed using SPSS version 19 (IBM Corp., Armonk, NY). Different types of birth injuries with outcome variables, maternal age, body mass index (BMI), mode of delivery, type of admission, duration of the 2nd stage of labor, birth weight, gender, shoulder dystocia, and presenting part at birth were independent variables. Data were expressed as mean ± standard deviation (SD) or percentages and analyzed by independent sample t-test and Mann-Whitney U test. The chi-square test or Fischer’s exact test was used to analyze the difference between categorical data. Statistical significance was accepted as p ≤ 0.05.

## Results

There were 51 birth injuries out of 27,854 deliveries. The birth injury rate was found to be 1.83 in 1000 births. Table 1 describes the maternal characteristics of our study population. The average maternal age was 29 years (mean ± SD, 29 ± 4.7) and BMI was 30.34 kg/m2 (30.34 ± 5.1) in 51 patients whose neonate suffered a mechanical birth injury. The gestational age was found to be 37 weeks (37.31 ± 2.02) and 10 out of those 51 patients (19.6%) were pre-term and the rest were term (>37 weeks), with the majority of patients being booked (90.2%). A total of 22 patients (43.1%) had some co-morbid condition, predominantly gestational diabetes, and preeclampsia.

**Table 1:** Demographic characteristics of mothers (n = 51) BMI: body mass index; GDM: gestational diabetes mellitus.

Variables	Categories	n (%)
BMI (kg/m^2^)	<25	6 (11.8%)
	25-30	22 (43.1%)
	>30	23 (45.1%)
Parity	Nulliparous	31 (60.8%)
	Multiparous	20 (39.2%)
Booking status	Booked	46 (90.2%)
	Un-booked	5 (9.8%)
Comorbid	Co-morbid (overall)	22 (43.1%)
	GDM	12 (54.5%)
	Preeclampsia	10 (45.4%)

While studying the characteristics of the mode of delivery, as shown in Table 2, we found that most patients underwent cesarean sections (64.7%), while 24% of the patients had spontaneous vaginal delivery and six patients (11.8%) had instrumental delivery. Out of a total of 33 cesarean sections, the majority were performed for emergency indications (72%). Various indications of cesarean sections and timing of delivery are mentioned in Table 2.

**Table 2:** Characteristics based on mode and timing of delivery (n = 51) SVD: spontaneous vaginal delivery; CS: cesarean section; CTG: cardiotocography.

Variables	Categories	n (%)
Mode of delivery (n = 51)	SVD	12 (23.5%)
	Instrumental	6 (11.8%)
	Cesarean section	33 (64.7%)
Indication of CS (n = 33)	Emergency	24 (72.7%)
	Elective	9 (27.3%)
Instrument type (n = 6)	Forceps	2 (28.6%)
	Vacuum	4 (57.1%)
Indications of CS (n = 33)	Failure to progress	16 (45.5%)
	Suspicious/pathological CTG	5 (15.2%)
	Previous scar	5 (15.2%)
	Breech	3 (9.1%)
	Placenta previa/accreta spectrum	3 (9.1%)
	Patient’s request	1 (3%)
Timing of delivery (n = 51)	Morning	26 (51%)
	Evening	13 (25%)
	Night	12 (24%)

**Figure 1:**
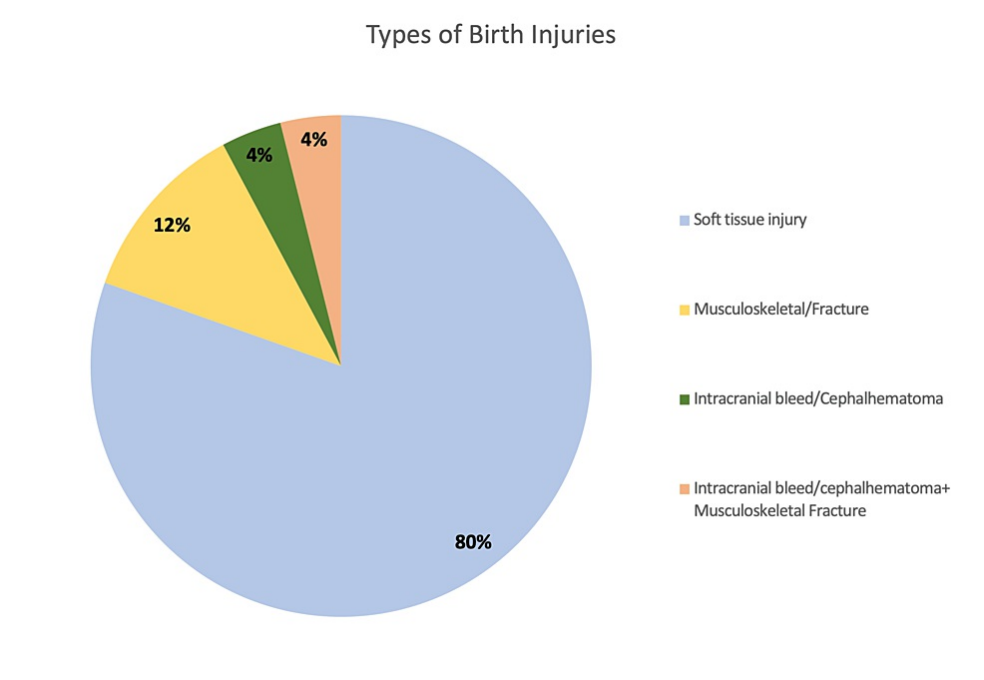
Type of birth injury (n = 51)

According to the classification of mechanical birth injuries (Figure 1), we found that 80% were soft tissue injuries, whereas 12% were musculoskeletal/fractures. There were two cases with intracranial bleed and another two cases were identified to have fractures along with intracranial bleed.

Out of total soft tissue injuries, most were lacerations (n = 34), abrasions (n = 4), and bruises (n = 2). In most of these cases, neonates were managed conservatively (n = 38), which required nothing more than the application of local antiseptics and oral analgesia. The exception was in two cases where the plastic surgery team was involved as the lacerations were deep and required suturing under local anesthesia. Table 3 mentions the details of all characteristics relevant to the fetus and neonates.

**Table 3:** Characteristics related to fetus (n = 51) ^§^ = These data are presented as n (%). ^*^ = These data are presented as (mean ± SD). APGAR: appearance, pulse, grimace, activity, and respirations; CTG: cardiotocography; GA: gestational age.

Variables	Categories	Value
APGAR score*	At 1 minute	7.75 ± 1.12
	At 5 minute	8.96 ± 0.20
Birthweight (kg)^§^	<2.5	7 (13.7%)
	2.5-3	28 (54.9%)
	3.1-3.5	16 (31.4%)
Fetal presentation^§^	Vertex	44 (86.3%)
	Breech	7 (13.7%)
Intrapartum fetal distress CTG^§^	Pathological	5 (9.8%)
	Suspicious	11 (15.7%)
Others^§^	Meconium-stained liquor	5 (9.8%)
	Oligohydramnios	7 (13.7%)
	Resuscitation after birth	4 (7.8%)
	Shoulder dystocia	5 (9.8%)
	Preterm (GA < 37 week)	10 (19.6%)
Birth weight (kg)*		2.84 ± 0.46
Head circumference*		33.68 ± 2.25

Table 4 summarizes the details of all mechanical injuries observed in our study. There was a total of seven clavicle fractures, four were isolated, one was compounded with humerus, and two were complicated with intracranial bleed. All were diagnosed with X-rays and six were delivered after shoulder dystocia. All the clavicle fractures were treated by sling or splint. One parietal depressed skull fracture happened during 2nd stage cesarean section and the baby was managed by craniotomy and shifted to NICU. Two babies had intracranial bleeding along with clavicle fractures. There was no mortality among all the mechanical birth injuries.

**Table 4:** Summary of description, investigation, and intervention according to birth injuries HIE: hypoxic-ischemic encephalopathy; EEG: electroencephalogram; MRI: magnetic resonance imaging; MRA: magnetic resonance arteriography; MRV: magnetic resonance venography; CT: computed tomography; NICU: neonatal intensive care unit; FFP: fresh frozen plasma.

Birth injuries with description	n	Investigation	Intervention
Soft tissue (n = 40)		None	Conservative/expectant (n = 38), suturing of laceration (n = 2)
Laceration	34		
Abrasions	4		
Bruises	2		
Musculoskeletal/fracture (n = 7)		X-rays (n = 6), CT brain (n = 1)	Arm sling (n = 2), splint (n = 3), NICU and then craniotomy (n = 1)
Clavicle fracture (isolated)	4		
Clavicle fracture + femur fracture	1		
Humerus fracture	1		
Parietal depressed skull fracture/subarachnoid hemorrhage	1		
Intracranial bleed/cephalhematoma (n = 2)		X-rays and CT (n = 1), X-rays and ultrasound (n = 1)	Observation (n = 2)
Subgaleal hematoma	1		
Cephalhematoma and abrasion	1		
Intracranial bleed/cephalhematoma + musculoskeletal fracture (n = 2)		EEG/MRI/MRA/MRV, coagulation profile, X-rays	FFP transfusion + intubation + antibiotics (n = 1), observed for fits, oxygen support in NICU, paracetamol (n = 1)
HIE + subgaleal hemorrhage + clavicular fracture	1		
HIE + left clavicular fracture + bruises on arms	1		

## Discussion

Our hospital, renowned as one of the largest tertiary care facilities nationwide, handles 5000+ deliveries annually and accepts referrals from across the country. We conducted this retrospective analysis to highlight the rate of mechanical birth injuries in our hospital along with various maternal and fetal factors related to it. Despite the large number of patients delivering in five years, the birth injury rate is found to be low, which is less than two per 1000 births.

This rate stands in contrast to research conducted in Kashan City, Iran, which reported a prevalence of 2.2% [9], as well as Ethiopia, where the rate was documented at 12.3% [10]. In South-East Nigeria, the birth injury rate was even higher, reaching 24% [11]. A study carried out in a public sector hospital in Pakistan in 2015 reported birth trauma in 148 out of 3,596 births, equating to a rate of 41.16 per 1,000 births [12]. Despite our study being conducted in a low- and middle-income country (LMIC), our findings indicated a significantly low rate of mechanical birth injuries compared to both other LMICs and studies conducted in Pakistan. Interestingly, our incidence of birth injuries is aligned with that of high-income countries (HICs). In the United States, for instance, these injuries are estimated to occur in approximately 2.6% of all births [13]. Similarly, a retrospective review of all births in Finland spanning from 1997 to 2017 showcased a decreasing incidence from 3.4% to 1.7% [14], mirroring our findings.

Our data have revealed a correlation between the mother's BMI and the occurrence of mechanical birth injuries in newborns. In our study, 45.1% of birth injuries were observed in patients with a BMI greater than 30 kg/m2, 43.1% in those with a BMI ranging from 25 to 30 kg/m2, and 11.8% in patients with a BMI below 25 kg/m2. Among the 51 cases of birth injuries, 12 patients (23.5%) had gestational diabetes mellitus (GDM). Interestingly, 11 of these patients (91.6%) had a BMI exceeding 25 kg/m2. However, due to the limited number of patients with birth injuries, we cannot definitively conclude a correlation between birth injuries, GDM, and elevated BMI in our study. Nonetheless, it is worth noting that most patients with GDM did have an increased BMI. This contrasts with a study conducted in Ethiopia, where birth injury rates were highest, approximately 70.8%, in patients with a normal BMI [15]. Our data reveal a correlation between birth injury rates and parity. Notably, primiparous women exhibited a higher incidence of birth injuries at 56.9%, compared to multiparous women at 43.1%. This pattern can be ascribed to the observation that pelvic joints and muscles in primiparous women tend to be more constricted than those in multiparous women, potentially resulting in increased pressure on the fetal presenting part during labor [5]. These findings align with other studies that similarly indicate a higher occurrence of instrumental delivery in nulliparous women [16] and increased rates of birth injuries in primigravida women when compared to multiparous women [5,15,17].

The presence of co-morbidities did not impact birth injury rates, except in the case of gestational diabetes. In our study, 12 out of 51 birth injuries were observed in patients with gestational diabetes, indicating clinical significance. However, due to the limited sample size, a direct correlation cannot be established. A study conducted by Tibebu et al. in 2023 emphasizes the clear association between gestational diabetes and mechanical birth injuries [17]. This association may be attributed to the fact that babies born to diabetic mothers often have macrosomia, leading to larger birth weights and an increased likelihood of birth injuries.

The mode of delivery significantly impacts both the type and incidence of birth injuries. Among the observed cases, 33 (64.7%) of birth injuries were identified in the cesarean section group, while 12 (23.5%) occurred during normal vaginal deliveries, and six (11.8%) were found in instrumental deliveries. These results diverge from other studies that have reported a higher incidence of birth injuries in instrumental deliveries compared to cesareans, with cesarean sections being identified as a protective factor against birth injuries [9,18,19].

When examining the indications for cesarean sections in our study, it becomes evident that most injuries are associated with emergency cesarean sections performed due to fetal distress, failure to progress in labor, fetal malpresentation in labor, low-lying placenta, and previous scar in labor. The predominance of injuries in these cases is likely due to the urgent nature of these cesareans, performed hastily by residents or instructors either for fetal or maternal indications to prevent further morbidity or mortality. This urgency can, at times, result in accidental or iatrogenic birth trauma. Interestingly, the types of birth injuries occurring in cesareans are predominantly soft tissue injuries. In contrast, those that occur after vaginal or instrumental delivery are mainly musculoskeletal, such as fractures or intracranial hematomas. No mortality has been observed due to birth injuries in our study. Out of the total, 40 cases (78.4%) involved soft tissue injuries like lacerations, bruises, and abrasions, all of which were treated conservatively, and the neonates were discharged. Accidental lacerations during cesarean section deliveries have been documented in 3% of the cases as reported in the study conducted in Italy. The study further highlighted a higher occurrence of accidental lacerations in emergent deliveries compared to elective cesarean deliveries [20], which is in conformity with the findings of our study.

Four cases of isolated clavicular fractures were identified, and one neonate was found to have a clavicle fracture along with a femur fracture on the same side. This occurred during delivery supervised by a senior resident with a consultant present. Furthermore, one case involved a humerus fracture due to shoulder dystocia, and another case had a parietal depressed skull fracture with subarachnoid hemorrhage. Two cases were further complicated by hypoxic-ischemic encephalopathy (HIE) along with clavicle fractures.

In our study, clavicular fractures were identified exclusively following normal vaginal deliveries, with a significant number of these cases being complicated by shoulder dystocia. One of the cases involved a neonate of average weight diagnosed with both a clavicle fracture and HIE. This neonate was delivered through normal vaginal delivery at term, and the delivery process was further complicated by shoulder dystocia. With an Apgar score of 8 at one minute and 9 at five minutes, the baby was initially diagnosed with a clavicle fracture and was transferred to the neonatal intensive care unit (NICU) for fracture management. However, on the third day of life, the baby's condition deteriorated, leading to intubation due to recurrent seizures. Subsequent investigations indicated mild HIE and the baby was later diagnosed with intracranial (subgaleal) hemorrhage, along with factor VII deficiency. The baby received care in the NICU and was discharged after a 10-day stay in the NICU.

Another significant case was where a clavicle fracture accompanied by HIE occurred in a large for gestational age (LGA) baby of a diabetic mother. The delivery was through spontaneous vaginal delivery (SVD) complicated by shoulder dystocia. Following delivery, the baby was transferred to the NICU, where a babygram revealed a left clavicle fracture. During the brief hospital stay, asphyxia markers indicated grade 1 HIE as well. Unfortunately, additional follow-up records were not available as the family chose to continue ongoing care at another healthcare facility.

In a particular case, a baby was diagnosed with a depressed skull fracture and intracranial hematoma. The delivery for this neonate involved an emergency cesarean section for a fully dilated mother due to the non-descent of the fetal head and pathological cardiotocography (CTG). Due to the second stage cesarean section, the fetal head became deeply impacted in the vagina, posing a challenge to the baby's delivery. An additional assistant successfully dislodged the fetal head using the "push-up" technique through the vagina. After delivery, the baby was promptly transferred to the NICU. After an X-ray and a CT scan of the head, a confirmed diagnosis revealed a depressed fracture of the skull, specifically in the right parietal bone. Neurosurgery was consulted, and the depressed skull fracture was surgically elevated. The neonate stayed in the NICU for a duration of two weeks following the surgery, after which the baby was discharged.

Various studies have investigated the influence of the time of delivery on birth injury rates. In our study, the majority of birth injuries occurred during the morning shift (0800-1700 hours), totaling 26 cases, followed by 13 injuries during the evening shift (1700-0000 hours) and 12 during the night shift (0000-0800 hours). When considering the total number of deliveries at our unit, 64% were carried out during the morning shift, 25% during the evening shift, and 11% during the night shift. This differs from a study conducted in 2023 by Tibebu et al. [17]. While numerous studies attribute the increased occurrence of birth injuries during nighttime hours to the lack of doctors, senior personnel, or sufficient staff, our study is in discordance with this pattern. We observed that most injuries occurred in the morning, despite the presence of more substantial support during those hours, indicating the absence of a specific relationship between the time of delivery and birth injuries. The intravenous administration of exogenous oxytocin is considered a high-alert medication due to its potential association with maternal, fetal, and neonatal morbidity [21]. Consequently, an alternative explanation for this trend could be attributed to the timing of deliveries, as most occur during the morning shift. Later in the day, unnecessary interventions like oxytocin augmentation and amniotomy may be avoided, as our institution requires the presence of an on-floor consultant during that time.

A significant number of birth injuries were observed in emergency cesarean cases. Enhancing surgical techniques during emergency situations, especially in scenarios involving instrumental delivery or cesarean section due to acute fetal bradycardia, is essential [22]. To achieve this improvement, the implementation of regular unit-based obstetric emergency drills, simulation training, refresher staff training, and supervised training of residents for surgical procedures is crucial. These comprehensive measures aim to fortify the preparedness and proficiency of healthcare teams in managing critical obstetric emergencies, ensuring improved outcomes for both mothers and newborns.

The strength of our study lies in its exemplary record-keeping practice and meticulous reporting of birth injuries within the hospital's Adverse Event Management System (AEMS). However, certain limitations must be acknowledged. The study is retrospective and confined to a single center over a narrow duration, limiting the generalizability of its findings to a broader population. The major limitation was that we did not have access to clinical records of brachial plexus injuries in neonates. Despite these limitations, the study's strengths in rigorous record-keeping contribute valuable insights into understanding and addressing birth injuries within the specified context and knowledge of associated obstetrical risk factors.

## Conclusions

The incidence of mechanical birth injuries in our study is notably lower when compared to studies conducted in other LMICs, attributed to our hospital's status as a prestigious training institute adhering to international quality accreditations like the Joint Commission International Accreditation (JCIA). Our practices prioritize one-on-one care during labor, meticulous fetal surveillance, and supervision by credentialed obstetricians. This clearly demonstrates that adhering to these standards can help maintain a lower birth injury rate. A higher BMI (>30 kg/m2) is associated with an increased risk of birth injury. We also found that parity, the presence of comorbidities, and the timing of delivery did not significantly impact the birth injury rate. Soft tissue injuries predominated, especially in emergency cesarean sections, whereas vaginal deliveries were more associated with fractures when augmented with oxytocin. Enhancing emergency teams and offering thorough hands-on training such as Practical Obstetric Multi-Professional Training (PrOMPT) to medical personnel caring for laboring mothers, particularly in critical scenarios like fetal distress or emergency cesareans, is crucial to ensure timely and safe interventions. Additionally, early recognition and counseling of families and caregivers regarding birth injuries are essential considerations for obstetricians and neonatologists.

## Appendices

**Table 5:** Association of BMI with birth injury. Sub-stratification based on the status of GDM (n = 51) BMI: body mass index; GDM: gestational diabetes mellitus.

GDM	Type of birth injury	N	BMI			p-value
			<25	25-30	>30	
With GDM	Soft tissue injury	9	1 (11.1%)	4 (44.4%)	4 (44.4%)	0.733
	Musculoskeletal/fracture	2	0	1 (50%)	1 (50%)	0.887
	Intracranial bleed/cephalhematoma + musculoskeletal fracture	1	0	0	1 (100%)	0.580
Without GDM	Soft tissue injury	32	2 (6.3%)	15 (46.9%)	15 (46.9%)	0.032
	Musculoskeletal/fracture	4	1 (25%)	1 (25%)	2 (50%)	0.634
	Intracranial bleed/cephalhematoma	2	2 (100%)	0	0	0.001
	Intracranial bleed/cephalhematoma + musculoskeletal fracture	1	0	1 (100%)	0	0.515

Proforma

Birth Injuries: Types and Associated Obstetric Risk Factors

Study ID: ________________________

Date of delivery:________________________

Gestational age at delivery: ­­­­­­­­­­­­________

Delivery conducted by: Consultant/Trainee Mode of delivery: Spontaneous vaginal delivery/Instrumental/Assisted

Cesarean section: Elective/Emergency

Indication: __________________

Instrumental delivery: Forceps/Vacuum Mid-cavity/Low-cavity/Outlet

Maternal Characteristics

BMI: _______

Age: _____________

Gravida: ____________ Duration of 2nd stage of labor: ____________________

Deep transverse arrest (DTA)/Persistent occiput posterior presentation (POP)? Onset of labor: Spontaneous/Induced or Elective C-section

Evidence of oligohydramnios?

Maternal co-morbid: ______________ *Newborn Characteristics*

APGAR score at one minute of life: ___________

APGAR score at five minutes of life: ___________

Birth weight: _____________________ Gender: Male/Female

Shoulder dystocia: Yes/No

Presenting part at birth: ___________

Fetal anomalies? _______________

**Table 6:** Characteristics of birth injury

Type of birth injury	Description	Investigation	Intervention	Disposition
Soft tissue injury				
Musculoskeletal/fracture				
Intracranial bleed/cephalhematoma				
Other				
